# Preclinical efficacy and dosimetry of CD44v6-directed terbium-161 radionuclide therapy

**DOI:** 10.7150/thno.135243

**Published:** 2026-08-24

**Authors:** Amanda Gustafsson, Anja Charlotte Lundgren Mortensen, Veronica Rondahl, Tianqi Xu, Klas Bratteby, Peter Bernhardt, Marika Nestor

**Affiliations:** 1 Department of Immunology, Genetics and Pathology, Science for Life Laboratory (SciLifeLab), Uppsala University, 752 37, Uppsala, Sweden.; 2 Department of Molecular Medicine and Surgery, Karolinska Institute, 171 77, Stockholm, Sweden.; 3 Department of Animal Biosciences, Swedish University of Agricultural Sciences, 750 07, Uppsala, Sweden.; 4 Department of Nuclear Medicine and Medical Physics, Karolinska University Hospital, 171 76, Stockholm, Sweden.; 5 Department of Oncology and Pathology, Karolinska Institute, 171 77, Stockholm, Sweden.; 6 Department of Medical Radiation Sciences, Institute of Clinical Sciences, Sahlgrenska Academy at University of Gothenburg, 413 45, Gothenburg, Sweden.; 7 Department of Medical Physics and Biomedical Engineering, Sahlgrenska University Hospital, 413 45, Gothenburg, Sweden.

**Keywords:** targeted radionuclide therapy, pancreatic ductal adenocarcinoma, squamous cell carcinoma, CD44v6, terbium-161

## Abstract

**Rationale:**

Lutetium-177 has demonstrated clinical success, particularly in neuroendocrine and prostate cancers, but disease recurrence and progression remain frequent. Terbium-161 offers improved therapeutic potential by delivering higher localized radiation doses. We have previously demonstrated preclinical efficacy of the CD44v6-targeted radiopharmaceutical [^177^Lu]Lu-AKIR001, which is currently under clinical investigation for multiple malignancies (NCT06639191). The present study explored the terbium-161-labeled analogue preclinically, focusing on pancreatic ductal adenocarcinoma, with early proof-of-concept investigations in squamous cell carcinoma, both being highly aggressive and treatment-resistant malignancies.

**Methods:**

Radioligand uptake was evaluated *in vitro* in squamous cell carcinoma and pancreatic ductal adenocarcinoma cell lines. *Ex vivo* biodistribution and dosimetry estimations confirmed a selective tumor uptake of [^161^Tb]Tb-AKIR001 before therapeutic efficacy was evaluated in both squamous cell carcinoma and pancreatic ductal adenocarcinoma xenograft models. Mice were administered [^161^Tb]Tb-AKIR001 of activities ranging from 4 to 10 MBq and compared to untreated controls. Potential adverse effects were evaluated by blood sampling, monitoring of body weights, and histopathology.

**Results:**

Specific, high-affinity binding of [^161^Tb]Tb-AKIR001 to CD44v6-positive cells was verified *in vitro*. Tumor uptake *in vivo* exceeded 30% injected activity per gram of tissue in the A431 model at 96 h post-injection. Peak tumor uptake in the BxPC3 model exceeded 150% injected activity per gram of tissue, with a mean absorbed dose to tumor of 17 Gy/MBq. Activity-dependent antitumor effects were observed in both xenograft models, accompanied by mild to moderate, transient hematologic effects that were significantly mitigated by activity fractionation. Histopathological evaluation revealed no treatment-related tissue damage in kidney, liver or spleen.

**Conclusion:**

[^161^Tb]Tb-AKIR001 demonstrated favorable biodistribution and dosimetry profiles, together with encouraging therapeutic effects without signs of severe toxicity. Compared to [^177^Lu]Lu-AKIR001, [^161^Tb]Tb-AKIR001 may offer added advantages in certain therapeutic contexts, and our results highlight [^161^Tb]Tb-AKIR001 as a promising candidate for further development in targeted radionuclide therapy.

## Introduction

Cancer therapy has entered a new era, shifting from one-size-fits-all strategies to more precise and personalized approaches. Among these, targeted radionuclide therapy (TRT) offers the unique ability to deliver cytotoxic radiation directly to cancer cells while reducing damage to surrounding healthy tissue. Unlike conventional external beam radiotherapy, TRT administers radiation internally via systemic or locoregional routes, facilitating selective targeting of malignant cells. By confining the ionizing radiation to the tumor site, off-target effects are reduced while therapeutic precision is enhanced. By combining the specificity of molecular targeting with the cytotoxic potential of radiation, TRT connects the gap between systemic and localized therapies, positioning it as a promising modality in the fight against both primary and metastatic malignancies 1,2.

Among the clinically approved radionuclides, lutetium-177 has gained prominence due to its favorable physical and chemical characteristics, such as its medium-energy beta emission and accompanying gamma radiation. Radiopharmaceuticals labeled with lutetium-177, for example [^177^Lu]Lu-DOTA-TATE (Lutathera^®^) and [^177^Lu]Lu-PSMA-617 (Pluvicto^®^), have demonstrated notable efficacy in treating neuroendocrine tumors and prostate cancer, respectively 3-5. Still, disease recurrence and progression remain frequent, most probably driven by tumor heterogeneity and micrometastatic deposits 3. Lutetium-177 is well-suited for irradiation of small tumors, but its mean tissue penetration range of approximately 0.7 mm (range 0.04 – 1.8 mm) limits its effectiveness against isolated tumor cells and small clusters, as the absorbed energy is often insufficient to induce cell death 3,6,7.

Terbium-161, a novel beta emitter with physical decay properties similar to lutetium-177, has the added advantage of emitting a higher number of low-energy conversion electrons (CE) and Auger-Meitner electrons (AE) per decay (Table 1) 3. CE and AE have higher linear energy transfer than beta electrons, enabling deposition of densely localized energy over a short distance 3,8. Thus, terbium-161 could potentially improve therapeutic outcomes in a range of tumor settings, including heterogeneous tumors as well as smaller lesions. While Monte Carlo simulations predict the greatest dosimetric advantage of terbium-161 in micrometastases and single tumor cells, the contribution of CE and AE remains substantial even in larger lesions (10 mm), where they account for approximately 25% of the absorbed dose compared with 10% for lutetium-177 6. Consistent with these predictions, preclinical studies have demonstrated enhanced therapeutic efficacy of [^161^Tb]Tb-PSMA-617 compared with [^177^Lu]Lu-PSMA-617 in prostate tumor-bearing mice at equivalent administered activities, as well as significantly reduced tumor establishment and growth when treatment was administered shortly after tumor inoculation 9. Similarly, L1CAM-targeting [^161^Tb]Tb-chCE7 demonstrated improved therapeutic efficacy compared with [^177^Lu]Lu-chCE7 at equitoxic doses (50% MTD) in subcutaneous xenografts of human ovarian carcinoma 10. These findings suggest that the enhanced local energy deposition of terbium-161 may be particularly advantageous in aggressive malignancies defined by high heterogeneity, early dissemination, and the presence of small metastatic lesions.

Building on our previous work with the novel CD44v6-targeting radiopharmaceutical [^177^Lu]Lu-AKIR001 and its ongoing phase I clinical evaluation (NCT06639191), we investigated the therapeutic potential of its terbium-161-labeled counterpart in two preclinical models 11,12. Primary focus was on pancreatic ductal adenocarcinoma (PDAC), with early investigations and proof-of-concept in squamous cell carcinoma (SCC), both being highly aggressive and treatment-resistant malignancies.

## Material and Methods

Material details can be found in Table S1.

### Cell lines

The human SCC cell line A431 was cultured in Dulbecco’s Modified Eagle Medium (DMEM), while the human PDAC cell line BxPC3 was cultured in Roswell Park Memorial Institute 1640 medium (RPMI-1640). Both cell culture media were supplemented with 10% fetal bovine serum and antibiotics (100 IU penicillin and 100 µg/mL streptomycin). Cells were cultured at 37 °C in a humidified atmosphere containing 5% CO_2_, being used for no longer than three months after thawing.

### AKIR001 antibody

AKIR001 is a full-length, human IgG1 anti-CD44v6 antibody 13. To minimize antibody-dependent cellular cytotoxicity and complement-dependent cytotoxicity, double LALA mutations have been incorporated. Antibody production and p-SCN-Bn-DOTA conjugation were done by GenScript ProBio (Nanjing, China), with an average chelator-to-antibody ratio of 1.8.

### Radioconjugate preparation and stability

[^161^Tb]TbCl_3_ was mixed with AKIR001 (10.4 µg/µl in a phosphate buffer with 0.04% polysorbate 80 (PS80), pH 6.5). To adjust reaction conditions, ammonium acetate (0.1 M, prepared in metal-free water and stored over chelex100) containing 50 mg/mL ascorbate was added, yielding a final pH of 5.0 – 5.5. Buffer-to-radionuclide volume ratio was approximately 5:1. The final mixture was incubated at 37 °C and 300 rpm for 30 min. Radiochemical yield was assessed by instant thin layer chromatography (ITLC) using citric acid (0.2 M, pH 5.5) as mobile phase and quantified with a high-speed image plate scanner (CR-35 Bio, Dürr Medical, Germany) and the software Aida Image Analyzer v.5.1 (Elysia-Raytest, Angleur, Belgium). A radiochemical yield above 95% was used for all experiments.

Radioconjugate stability (37 °C, 300 rpm) was evaluated by ITLC in phosphate-buffered saline (PBS), mouse serum diluted 1:1 with PBS, or in PBS with the presence of a 500-fold molar excess of ethylenediaminetetraacetic acid (EDTA).

High-performance liquid chromatography (HPLC) was performed for additional analysis of [^161^Tb]Tb-AKIR001 stored in PBS. After a 1:15 dilution of [^161^Tb]Tb-AKIR001, 20 µL sample was injected on a size exclusion column (TSKgel G3000SWXL, 7.8 mm I.D. x 30 cm, 5 µm) equipped with a guard column (TSKgel SWXL, 6 mm I.D.x 4 cm, 7 µm) on an HPLC system (Shimadzu, LC-20 Prominence). The analysis was run with an isocratic method (10% acetonitrile in PBS) at 1.0 mL/min for 20 min. Samples were analyzed at 280 nm (Ch 1.), 254 nm (Ch 2.), and a radiodetector (Flow-Count) for the radioactive signal. The delay between the UV detector and the radio detector at this flow rate is estimated to approximately 0.65 min.

### Binding specificity assays

Binding specificity of [^161^Tb]Tb-AKIR001 was determined with cell-based competition assays. Briefly, 3 – 5 × 10^4^ BxPC3 cells were seeded in 48-well plates 48 h before addition of 10 nM (>10 × K_D_) [^161^Tb]Tb-AKIR001, either alone or together with a 100-fold molar excess of unlabeled AKIR001. 24 h later unbound [^161^Tb]Tb-AKIR001 was removed, and cells were washed with PBS before being harvested and counted (TC20^TM^ Automated Cell Counter, BioRad, Sweden). Subsequent cell-associated radioactivity was quantified using a gamma counter (2480 Wizard 2, Wallace, Finland). Binding specificity of AKIR001 to A431 has been evaluated in previous studies 11.

### LigandTracer real-time binding measurements

Antibody-antigen interactions were monitored in real-time with LigandTracer (Ridgeview Instruments AB, Uppsala, Sweden). Approximately 0.75 – 1 × 10^6^ A431 or BxPC3 cells were seeded onto tilted 10 cm cell culture dishes and incubated until attachment, typically overnight. The culture medium was then exchanged for 10 mL of fresh medium, followed by a further 24 h incubation. Binding experiments were performed at room temperature using either LigandTracer Grey or White according to manufacturer’s instructions. Briefly, a baseline signal was established in ligand-free medium, after which [^161^Tb]Tb-AKIR001 was added stepwise at concentrations of 0.3 nM and 1 nM to assess association. Dissociation was initiated by replacing the radioactive medium with fresh, ligand-free medium. Data were analyzed according to manufacturer’s instructions using TraceDrawer 1.9.2 (Ridgeview Instruments AB, Uppsala, Sweden), fitted to a standard 1:1 binding model.

### Xenograft establishment

A431 and BxPC3 xenografts were established in female BALB/c nu/nu mice (Charles River Laboratories, MA, USA or Janvier Labs, Le Genest-Saint-Isle, France) by subcutaneous (s.c.) injection of approximately 1 × 10^7^ cells suspended in serum-free medium into the posterior right flank. Animals were maintained under standard laboratory conditions with tumor growth, body weight, and general condition monitored multiple times per week. Tumor dimensions were measured with a digital caliper (Mitutoyo, Sweden), and volumes calculated as (length × width × depth) × 0.52 for A431 xenografts, or (length × width^2^) × 0.5236 for BxPC3 xenografts. Animals were euthanized via intraperitoneal (i.p.) injections of ketamine and xylazine, followed by cardiac puncture. Humane endpoints were defined as tumor volume exceeding 1000 mm^3^ and/or body weight loss of more than 10% compared to the maximum recorded weight. All procedures were conducted in accordance with Swedish legislation and were approved by the Uppsala Committee of Animal Research Ethics (ethical permit numbers 5.8.18-10966/2020 and 5.8.18-05467/2025).

### *Ex vivo* biodistribution of [^161^Tb]Tb-AKIR001

Biodistribution of [^161^Tb]Tb-AKIR001 was determined *ex vivo* in xenografted mice. Tumors and organs were excised, weighed, and analyzed with a gamma counter to quantify decay-corrected organ-associated radioactivity.

Mice (age = 12 weeks; weight = 19.7 ± 1.3 g, mean ± s.d.) bearing A431 xenografts (152.9 ± 86.3 mm^3^, mean ± s.d.) were injected with 195 kBq/15 µg [^161^Tb]Tb-AKIR001 in 100 µl PBS i.v. in the tail vein on day eight post-inoculation. Biodistribution was evaluated at 4 h (N = 4), 24 h (N = 4), 48 h (N = 4), 96 h (N = 7), 168 h (N = 3), and 240 h (N = 4) post-injection (p.i.). Three animals in the 96-h cohort received an additional 450 µg unlabeled AKIR001 to block uptake.

Similarly, mice (age = 7 weeks; weight = 17.63 ± 1.50 g, mean ± s.d.) bearing BxPC3 xenografts (28.0 ± 10.2 mm^3^, mean ± s.d.) were injected with 300 kBq/15 µg [^161^Tb]Tb-AKIR001 in 100 µl PBS via intravenous (i.v.) tail vein injection on day 7 post-inoculation.

Biodistribution was assessed at 4 h (N = 5), 24 h (N = 5), 48 h (N =5), 96 h (N = 4), and 168 h (N = 5) p.i.

### Dosimetry estimations

Organ dosimetry was calculated for [^161^Tb]Tb-AKIR001 using the BxPC3 *ex vivo* biodistribution data from the present study, complemented with [^177^Lu]Lu-AKIR001 data from a previous study 12. Bi-exponential curves (with three parameters) were fitted to activity concentration versus time data. The biokinetics of the terbium-161 compound were assumed to be the same as for the lutetium-177 compound. Fits were performed for all possible data point combinations and integrated to infinity to obtain time-integrated activity concentrations (TIACs). Absorbed energy per decay was primarily derived from the MOBY mouse phantom. For organs not included in MOBY, estimates were made via Monte Carlo simulations in spheres matching the organ masses. Bone marrow activity concentration was assumed to be 36% of that in blood. Absorbed doses were then calculated by multiplying TIACs with the corresponding absorbed energy for each tissue.

### Therapeutic effect of [^161^Tb]Tb-AKIR001

Therapeutic effect of [^161^Tb]Tb-AKIR001 (50 µg in 100 µL PBS, administered i.v. via the tail vein) was evaluated in BALB/c nu/nu mice bearing A431 or BxPC3 xenografts.

A431 xenograft-bearing animals (age = 12 weeks; weight = 19.8 ± 1.3 g, mean ± s.d.) were randomized into four groups: PBS control (N = 4), 5 MBq [^161^Tb]Tb-AKIR001 (N = 10), 7.5 MBq [^161^Tb]Tb-AKIR001 (N = 5), and 10 MBq [^161^Tb]Tb-AKIR001 (N = 5). Treatment was initiated when the average tumor volume was 255.2 ± 65.5 mm^3^ (mean ± s.d.) at day eight post-inoculation. Tumor volumes at treatment start were comparable between groups (p > 0.05, Figure S1A).

BxPC3 xenograft-bearing animals (age = 8 weeks; weight = 18.0 ± 1.2 g, mean ± s.d.) were similarly randomized into four groups: PBS control (N = 8), 4 MBq [^161^Tb]Tb-AKIR001 (N = 8), 8 MBq [^161^Tb]Tb-AKIR001 (N = 8), and fractionated 8 MBq [^161^Tb]Tb-AKIR001 (N = 8) administered as two injections of 4 MBq one week apart. A group of tumor-free littermate controls (N = 3) was kept alongside treated mice for reference. Treatment was initiated when tumors had reached an average volume of 73.1 ± 32.1 mm^3^ (mean ± s.d.) at day nine post inoculation. Tumor volumes at treatment start were comparable between groups (p > 0.05, Figure S1B).

Treatment-related hematologic toxicity was monitored by serial blood sampling (tail vein, 20 µL), with analysis of white blood cells, red blood cells, platelets, and hemoglobin performed using a veterinary hematology analyzer (Exigo H400, Boule, Sweden). In the A431 model four to five animals per TRT-treated group were sampled on days 0, 15, 23, and 35. In the BxPC3 model, blood sampling was performed in a staggered manner during the first 15 days. Animals were alternately sampled on days 0, 5, 10, and 15 to minimize repeated stress and potential related effects on hematologic parameters. Thereafter, all animals were sampled on days 21, 35, and 56.

Study endpoints were set to 90 days post-treatment for A431 xenografts, and 100 days post-treatment for BxPC3 xenografts. Within each treatment group, animals were classified according to their treatment response: complete responders (complete tumor remission), stable disease survivors (tumors present but still alive at the study endpoint), partial responders (tumor growth delayed relative to controls, but maximal tumor size reached before clinical endpoint), early removal (euthanized due to toxicity or ulceration), and non-responders (no measurable treatment effect).

### SPECT Imaging

Single-photon emission computed tomography/computed tomography (SPECT/CT) was performed using a small-animal nanoScan SPECT/CT (Mediso Medical Imaging Systems, Hungary). One mouse from each treatment group in the BxPC3 model was imaged at 96 h p.i.

Mice were anesthetized with 3% sevoflurane and placed on a pre-heated scanner bed to prevent hypothermia. The whole-body CT scan was acquired for 5 min with a semi-circle field of view (FOV), 480 projections, X-ray of 50 kVp and 620 μA, and 1:4 binning. The SPECT scan was performed in the same range as the CT scan under the Terbium-161 window (48 keV, 25.65 keV, and 74.75 keV) with an acquisition frame of 7 s (8 MBq [^161^Tb]Tb-AKIR001) or 10 s (4 MBq and 2 × 4 MBq [^161^Tb]Tb-AKIR001), resulting in a total scanning time of 8 min and 11 min, respectively. The CT raw files were reconstructed using Filter Back Projection in Nuclide 2.03 Software (Mediso Medical Imaging Systems Ltd.). The SPECT acquisition data were reconstructed using the Nuclide 2.03 software and Tera-Tomo™ 3D SPECT reconstruction technology (Mediso Medical Imaging Systems Ltd.) with 3 subsets and 48 iterations. The SPECT and CT DICOM files were fused and analyzed using Tera-Tomo™ 3D SPECT reconstruction technology (Mediso Medical Imaging Systems Ltd.). The SPECT images are presented as maximum intensity projections (MIPs) in the RGB color scale.

### Histopathological evaluation

At euthanasia, the kidneys, liver, spleen and tumors (when macroscopically identifiable) were collected from BxPC3 tumor-bearing mice. Tumors and organs from BxPC3 xenograft-bearing mice treated with [^177^Lu]Lu-AKIR001 were obtained from a previous study 12. All tumors and organs were fixed in 4% formaldehyde for 24 h and stored in 70% ethanol until trimming. The organs were trimmed according to recommendations 14-16: kidney, two sections, one transverse and one longitudinal; liver, three sections, one from the left lateral lobe, one from the left and right medial lobe including gall bladder, and one from the caudate lobe; spleen, one longitudinal section. Tumors were trimmed to one to three sections consisting of one transverse across the widest diameter of the sample, and when possible (depending on the size of the sample) two edges perpendicular to the transverse section. Trimmed samples were processed in an automated tissue processing machine (Excelsior AS, Thermo Scientific) for dehydration, followed by paraffin-embedding, microtome sectioning (4 μm), and stained with Mayer’s hematoxylin and eosin using a standard protocol.

The sections were analyzed and scored by a veterinary anatomic pathologist experienced in laboratory animal pathology, blinded to the specific treatment of the samples. The scoring of kidney, liver and spleen sections was performed semi-quantitatively as described by Mann et al. 17, with lesion definitions as described by Willard-Mack et al., Frazier et al., and Thoolen et al. 18-20. In brief, tissue with an appearance within normal limits, considering the age, sex, and mouse strain, was given a score of 0. Lesions with minimal change, i.e., the amount of change barely exceeded change considered to be within normal limits, were given a score of 1. Lesions with mild change, i.e., the lesion was easily identified but of limited severity, were given a score of 2. Lesions with moderate change, i.e., the lesion was prominent but there was significant potential for increased severity, were given a score of 3. Lesions with severe change, i.e., the degree of change occupied the majority of the organ, were given a score of 4.

### Data visualization and statistical analysis

The number of technical replicates is specified as “n”, and biological replicates as “N”.

GraphPad Prism (GraphPad Software, CA, USA) for Mac was used for data analysis and visualization, unless otherwise specified. Statistical analyses were performed with p < 0.05 being significant. To specify the level of significance, asterisks were used with * corresponding to p < 0.05, ** < 0.01, *** < 0.001, and **** < 0.0001.

For specificity assays, t-tests determined significance between blocked and non-blocked conditions. Mean tumor volumes at treatment start and hematologic parameters were analyzed by one-way ANOVA with Tukey’s multiple comparison test. Biokinetic profiles were analyzed using two-way ANOVA with time and treatment as factors, followed by Šídák's multiple comparison test. Overall survival was evaluated with Kaplan-Meier survival analysis and compared using the log-rank (Mantel-Cox) test. Histopathological lesion scores for each organ were compared between groups using the Kruskal-Wallis test. For parameters showing a significant overall group effect (p < 0.05), Dunn’s multiple-comparison test was applied for pairwise group comparisons.

## Results

### [^161^Tb]Tb-AKIR001 remains stable in the presence of ascorbate

Stability of [^161^Tb]Tb-AKIR001 after radiolabeling was evaluated by ITLC in either PBS, mouse serum, or in PBS with a presence of a 500-fold molar excess of EDTA (Table 2). Experiments were done in triplicates. The radioconjugate remained stable for at least 24 h (> 95% yield radiochemical purity, RCP). Additional HPLC analysis in PBS indicated high antibody stability (> 97% RCP) for at least 48 h in presence of 50 mg/mL ascorbate (Table 3).

### [^161^Tb]Tb-AKIR001 binds specifically to CD44v6 with high affinity

**Figure 1:**
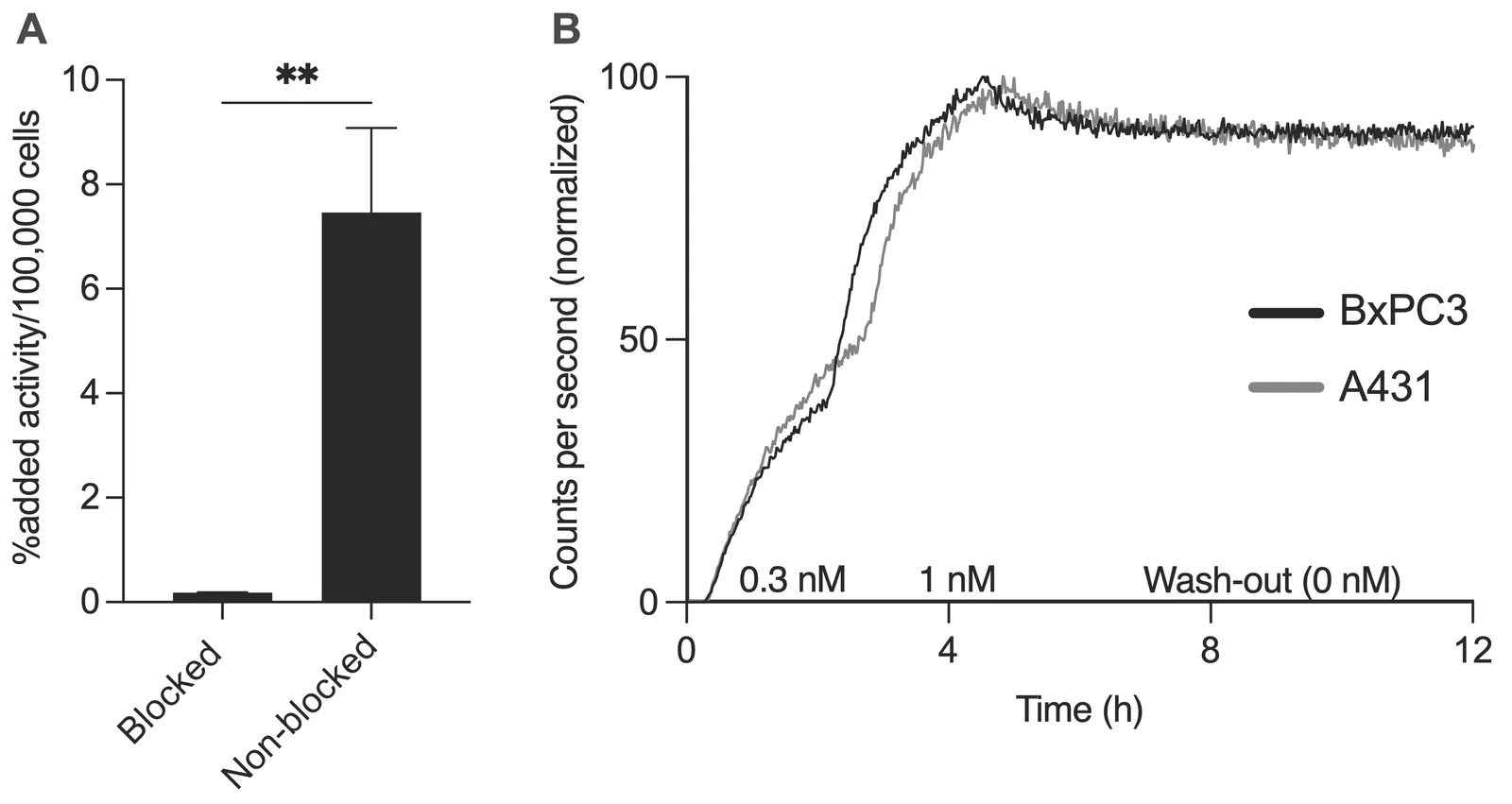
***In vitro* characterization of [^161^Tb]Tb-AKIR001.** (A) [^161^Tb]Tb-AKIR001 binds specifically to BxPC3 (n = 4, N = 3). (B) Representative curves from LigandTracer real-time binding measurements with BxPC3 (n = 1, N = 3) and A431 cells (n = 1, N = 1). Number of * indicates level of significance. Error bars represent s.d.

Binding of [^161^Tb]Tb-AKIR001 to CD44v6 was verified *in vitro* by cell-based competition assays and LigandTracer real-time binding measurements. CD44v6-specific binding to BxPC3 cells was demonstrated (n = 4, N = 3, Figure 1A), together with a high apparent affinity to both A431 (n = 1, N = 1, Figure 1B & Table 4) and BxPC3 cells (n = 1, N = 3, Figure 1B & Table 4).

### Excellent tumor targeting and high absorbed dose to tumor

**Figure 2:**
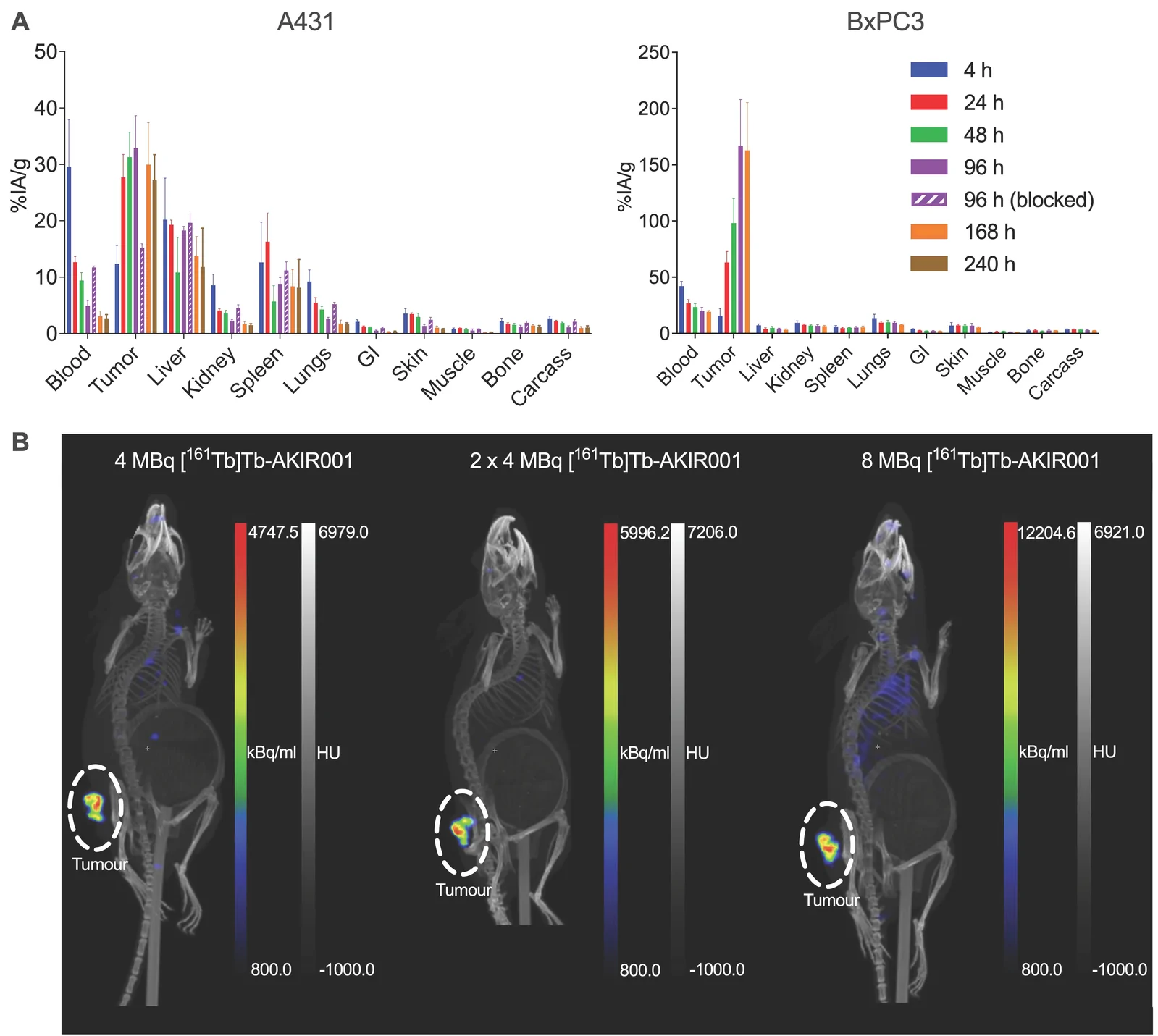
** Biodistribution of [^161^Tb]Tb-AKIR001.** (A) Mice bearing A431 xenografts were injected with 195 kBq/15 µg [^161^Tb]Tb-AKIR001 and dissected at 4 h (N = 4), 24 h (N = 4), 48 h (N = 4), 96 h (N = 7), 168 h (N = 3), and 240 h (N = 4) p.i. Three animals in the 96-h cohort received an additional 450 µg unlabeled AKIR001 to block uptake. Mice bearing BxPC3 xenografts were injected with 300 kBq/15 µg [^161^Tb]Tb-AKIR001 and dissected at 4 h (N = 5), 24 h (N = 5), 48 h (N = 5), 96 h (N = 4), and 168 h (N = 5) p.i. Data presented as mean % injected activity per gram (IA/g) of tissue. Error bars represent s.d. (B) BxPC3 tumors visualized via SPECT/CT imaging at 96 h p.i. of 4 MBq, 2 × 4 MBq (first injection), and 8 MBq [^161^Tb]Tb-AKIR001.

To confirm *in vivo* binding specificity and selectivity, *ex vivo* biodistribution was performed in A431 and BxPC3 xenograft models. In the A431 model, peak tumor uptake of [^161^Tb]Tb-AKIR001 exceeded 30% injected activity per gram (%IA/g) of tissue (Figure 2A & Table S2), while in the BxPC3 model, peak tumor uptake exceeded 150% IA/g of tissue (Figure 2A & Table S3). In the A431 model tumor uptake was successfully reduced after administration of excess unlabeled AKIR001 (Figure 2A). SPECT/CT imaging of mice bearing BxPC3 xenografts further confirmed tumor uptake and off-target clearance (Figure 2B).

From the BxPC3 *ex vivo* data, the estimated mean absorbed dose to the tumor was calculated to be 17 Gy/MBq (Table 5). Corresponding estimates for [^177^Lu]Lu-AKIR001 in the same tumor model were based on data from a previous study 12. Organ-specific biokinetics were compared between the radionuclides, with details provided in the supplemental material (Figure S2). Overall, biodistribution profiles were comparable, with statistically significant differences observed in selected organs and/or at individual time points. Blood activity was consistently higher for terbium-161, with significant differences at 24 h and 168 h post-injection. Importantly, comparable uptake profiles were observed for tumor and bone, whereas uptake in liver, spleen, and kidney differed only at the final time point.

### [^161^Tb]Tb-AKIR001 improves survival and fractionation reduces hematologic toxicity

Therapeutic efficacy of [^161^Tb]Tb-AKIR001 was investigated in BALB/c nu/nu mice bearing A431 or BxPC3 xenografts.

**Figure 3:**
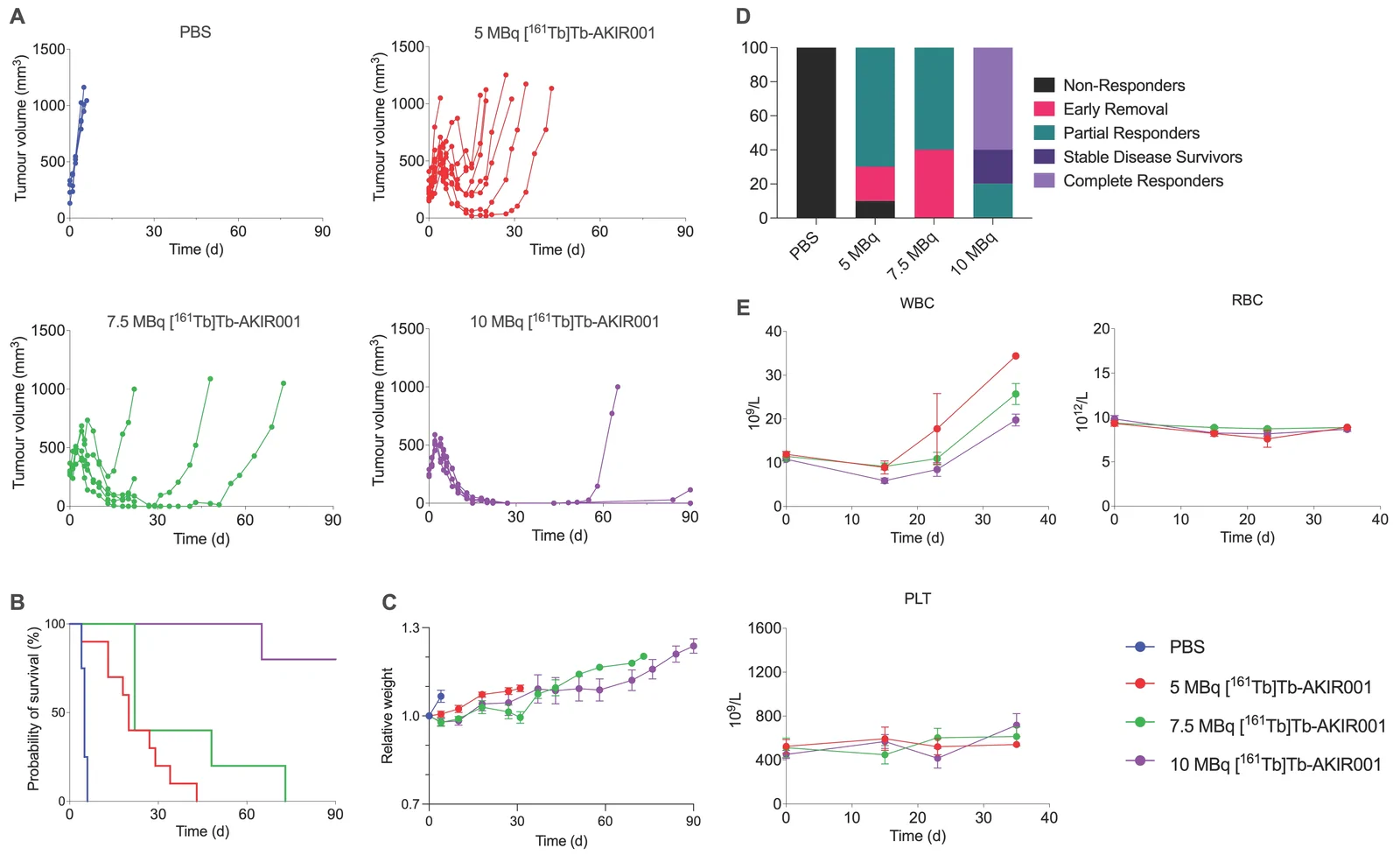
** Therapeutic effect of [^161^Tb]Tb-AKIR001 in A431 xenografts.** Mice bearing A431 xenografts were randomized into four groups: PBS control (N = 4), 5 MBq [^161^Tb]Tb-AKIR001 (N = 10), 7.5 MBq [^161^Tb]Tb-AKIR001 (N = 5), and 10 MBq [^161^Tb]Tb-AKIR001 (N = 5). Data presented as (A) individual tumor growth, (B) survival, (C) relative body weight, and (D) distribution of animals by treatment group and response category: non-responders (no measurable treatment effect), early removal (euthanized due to toxicity or ulceration), partial responders (tumor growth delayed relative to controls, but tumor-baring limit reached), stable disease survivors (tumors present but still alive at day 90), complete responders (complete tumor remission). (E) Hematologic parameters were monitored by blood sampling. WBC, white blood cells; RBC, red blood cells; PLT, platelets. Data in (C) and (E) are presented as group mean ± s.e.m.

In the A431 proof-of-concept study, animals received 5, 7.5, or 10 MBq [^161^Tb]Tb-AKIR001, or PBS as control. Activity-dependent antitumor effects were observed, with the strongest response after 10 MBq [^161^Tb]Tb-AKIR001 (Figure 3A-B, D & Table 6). Body weights remained stable throughout the study (Figure 3C), and hematologic monitoring of treated animals did not indicate treatment-related hematologic toxicity (Figure 3E).

**Figure 4:**
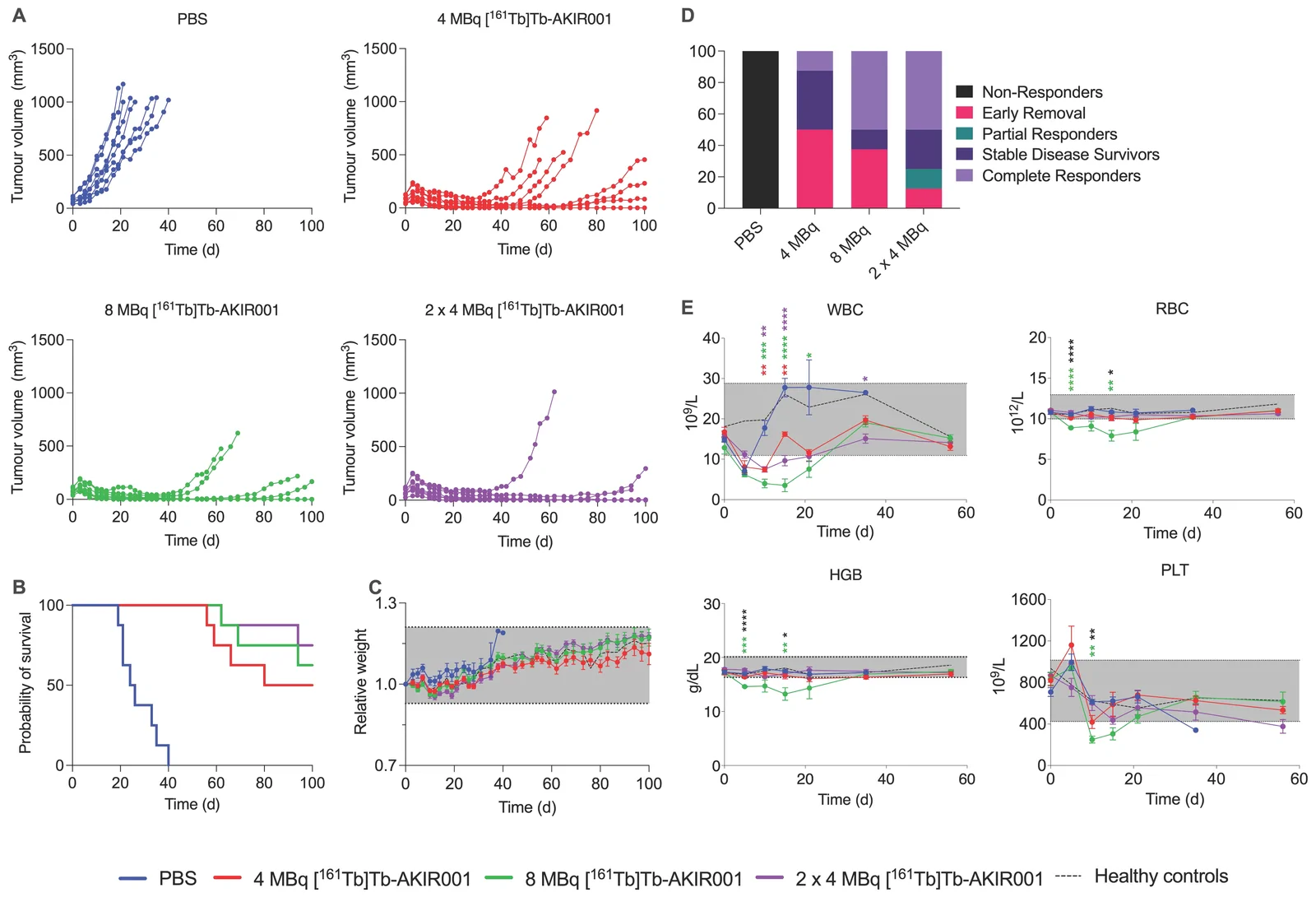
** Therapeutic effect of [^161^Tb]Tb-AKIR001 in BxPC3 xenografts.** Mice bearing BxPC3 xenografts were randomized into four groups: PBS control (N = 8), 4 MBq [^161^Tb]Tb-AKIR001 (N = 8), 8 MBq [^161^Tb]Tb-AKIR001 (N = 8), and 8 MBq [^161^Tb]Tb-AKIR001 given one week apart in two fractions of 4 MBq each (N = 8). Data presented as (A) individual tumor growth, (B) survival, (C) relative body weight, and (D) distribution of animals by treatment group and response category: non-responders (no measurable treatment effect), early removal euthanized due to toxicity or ulceration, partial responders (tumor growth delayed relative to controls, but tumor-baring limit reached), stable disease survivors (tumors present but still alive at day 100), complete responders (complete tumor remission). (E) Hematologic parameters were monitored by serial blood sampling. Number of * indicate degree of significance when detected compared to both PBS and healthy controls. Black * indicates significant difference between 8 MBq and 2 × 4 MBq. The shaded area represents the range of littermate controls. WBC, white blood cells; PLT, platelets, RBC, red blood cells; HGB, hemoglobin. Data in (C) and (E) are presented as group mean ± s.e.m.

In the BxPC3 study, animals were administered 4 MBq, 8 MBq, fractionated 2 × 4 MBq [^161^Tb]Tb-AKIR001, or PBS as control. Three tumor-free healthy littermate controls were kept alongside treated mice for reference. Treatment resulted in activity-dependent antitumor effects, with 50% of mice treated with 8 MBq [^161^Tb]Tb-AKIR001 achieving complete remission (Figure 4A-B, D & Table 6). Compared with the previous [^177^Lu]Lu-AKIR001 study in the same BxPC3 xenograft model 12, [^161^Tb]Tb-AKIR001 showed improved therapeutic performance at matched administered activity. Survival after 4 MBq [^161^Tb]Tb-AKIR001 was significantly prolonged compared with 4 MBq [^177^Lu]Lu-AKIR001 (p = 0.002). In addition, survival after 4 MBq, 8 MBq, or fractionated 2 × 4 MBq [^161^Tb]Tb-AKIR001 did not differ significantly from survival after 12 MBq [^177^Lu]Lu-AKIR001(p = 0.28, p = 0.51, and p = 0.85, respectively).

Body weights remained stable across groups (Figure 4C). A transient reduction in white blood cell counts was observed after [^161^Tb]Tb-AKIR001 treatment, most pronounced after a single-dose 8 MBq administration (Figure 4E). Fractionated administration of the same total activity, 2 × 4 MBq, significantly reduced hematologic effects compared with single-dose 8 MBq treatment (p < 0.05), while maintaining comparable survival benefit (p = 0.59).

### Histopathological evaluation revealed no toxic effects

The histopathological evaluation of kidney, liver, and spleen from the BxPC3 xenograft-bearing mice treated with [^161^Tb]Tb-AKIR001 or [^177^Lu]Lu-AKIR001, and from control mice from both studies showed no toxic effects (Figure S3-6 & Supplementary Excel file). As identical findings, three mice were found to have tumors of hematopoietic origin in the spleen: two with histiocytic sarcoma and one with follicular lymphoma. These tumors occur as spontaneous background tumors in BALB/c mice 21, although the prevalence in BALB/c nu/nu is not reported. These were excluded from the spleen scoring data. There were no differences between [^161^Tb]Tb-AKIR001 and [^177^Lu]Lu-AKIR001 in neoplastic cell differentiation or estimated area of necrosis within the examined tumor sections (Figure S7-8 & Supplementary Excel file).

## Discussion

This preclinical study evaluated terbium-161 for CD44v6-TRT using the novel AKIR001 antibody and investigated its potential as an alternative or complement to lutetium-177. Previously, [^177^Lu]Lu-AKIR001 demonstrated promising therapeutic efficacy preclinically and is currently under clinical investigation (NCT06639191) 11,12. Clinical experience with terbium-161 remains limited but encouraging, including first-in-human applications of [^161^Tb]Tb-DOTATOC, [^161^Tb]Tb-PSMA-617, and the non-internalizing [^161^Tb]Tb-LM3 7,22-24.

[^161^Tb]Tb-AKIR001 demonstrated improved therapeutic efficacy compared with [^177^Lu]Lu-AKIR001 in two xenograft models. In A431 xenografts, 10 MBq [^161^Tb]Tb-AKIR001 produced responses comparable to 15 MBq [^177^Lu]Lu-AKIR001 (Figure 3) 11. In the BxPC3 model, 4 MBq [^161^Tb]Tb-AKIR001 significantly prolonged survival compared to 4 MBq [^177^Lu]Lu-AKIR001 (p = 0.002), and 50% (4/8) of mice achieved complete remission after either a single 8 MBq administration or two fractionated 4 MBq injections (Figure 4). By comparison, 12 MBq [^177^Lu]Lu-AKIR001 previously yielded 40% (2/5) complete remission in the same xenograft model 12. These findings agree with the nearly 1.5-fold higher tumor absorbed dose predicted for [^161^Tb]Tb-AKIR001 (Table 5) and theoretical dosimetry estimates suggesting that 27-29% lower activity of terbium-161 may be sufficient to achieve tumor absorbed doses equivalent to lutetium-177 25.

Some aspects of the dosimetric comparison should however be interpreted with caution. The BxPC3 tumors were small at dissection, and although *ex vivo* gamma counting avoids imaging partial-volume effects, small masses may increase the relative uncertainty in weighing, dissection, and %IA/g estimates. Early, small xenografts may also differ from larger tumors in perfusion and necrotic fraction. Thus, tumor uptake and absorbed dose estimates should be interpreted in the context of small BxPC3 xenografts. Additionally, comparisons with [^177^Lu]Lu-AKIR001 relied on previous data rather than a strict head-to-head experiment, and differences in tumor size, animal weight, and injected activity may influence the comparison. Nevertheless, the relatively uniform terbium-161/lutetium-177 absorbed dose ratio across tissues suggests that the calculated increase is mainly driven by the higher emitted electron energy per decay of terbium-161, under the assumption of similar biokinetics.

Although macroscopic dose distributions of terbium-161 and lutetium-177 are comparable, terbium-161 emits a substantially higher proportion of short-range AE and CE, increasing local energy deposition close to the decay site 6. Therapeutic efficacy may therefore be enhanced through intracellular irradiation following internalization and/or through membrane-proximal irradiation from non-internalized, membrane-bound radioligand 26. While AKIR001 shows limited internalization *in vitro*, increased tumor uptake and decreased blood activity over time suggest prolonged tumor retention *in vivo* 12. Internalization may also be slow or delayed, as seen for DOTA-LM3 27. Thus, the therapeutic efficacy may reflect contributions from both intracellular and membrane-proximal irradiation, consistent with studies demonstrating particularly large therapeutic gains for non-internalizing constructs such as [^161^Tb]Tb-DOTA-LM3 28. In contrast, Spoormans et al. observed only modest absorbed dose increases when substituting lutetium-177 with terbium-161 in a setting where the radioligand lacked nuclear localization 29.

Microdosimetric modelling predicts the dose enhancement associated with terbium-161 relative to lutetium-177 to increase as target size decreases, reaching factors of 2 – 3 at the cellular and micrometastatic scale 6,8. However, biological impact at the cellular scale depends strongly on tumor microarchitecture, receptor distribution, internalization, and subcellular localization. Since nuclear localization of AKIR001 has not been demonstrated, the macroscopic dose increase should not be interpreted as a direct estimate of nuclear or cellular dose enhancement. Nonetheless, the improved therapeutic efficacy observed suggests that additional short-range electrons from terbium-161 confer a biological advantage even without confirmed nuclear localization.

Substituting lutetium-177 with terbium-161 increased absorbed dose per MBq to both tumors and normal tissues (Table 5), which may have implications for maximum tolerated dose. Thus, the therapeutic benefit depends on whether tumor targeting, clearance, and treatment scheduling provide a sufficient therapeutic window. The present study was not designed for maximum tolerated dose determination, and dedicated studies are required for clinical translation. Within the investigated activity range, hematologic toxicity was the main limiting factor, while fractionated administration mitigated these effects without compromising efficacy (Figure 4B & E). Furthermore, there were no toxic injuries found in the kidneys, liver, or spleen at time of euthanasia, which also supports that the hematologic toxicity was transient and reversible (Figure S3-6 & Supplementary Excel file).

Bone marrow toxicity remains a central concern in TRT and was a key focus of the present evaluation. Although the estimated bone marrow dose was approximately 1.4-fold higher for [^161^Tb]Tb-AKIR001 than for [^177^Lu]Lu-AKIR001 (Table 5), hematologic toxicity remained mild to moderate and transient (Figure 3E & Figure 4E) 12. Fractionating 8 MBq [^161^Tb]Tb-AKIR001 into two 4 MBq injections significantly reduced toxicity without compromising efficacy (Figure 4E), consistent with clinical experience using fractionated [^177^Lu]Lu-J591 30,31. This supports fractionation as a strategy to reduce peak blood activity. Additionally, dosimetry modelling of the CD44v6-targeting antibody BIWA4 predicted bone marrow absorbed doses for terbium-161 to be comparable to rhenium-186, while lutetium-177 yields lower marrow doses 32. The acceptable safety profile of [^186^Re]Re-BIWA4 further supports feasibility for managing marrow-related toxicity from terbium-161 33.

A key limitation is the lack of cross-reactivity between AKIR001 and murine CD44v6, preventing assessment of target-mediated uptake and toxicity in normal CD44v6-expressing tissues such as skin, cervix, cornea, and tonsils 34. This is particularly relevant since dosimetry modelling predicts substantially higher absorbed doses to the basal skin layer with terbium-161 compared to lutetium-177 (23.7 vs. 9.5 Gy GBq^-1^) 32. Future studies should therefore include models that enable evaluation of target-expressing normal tissues and potential associated toxicity.

## Conclusion

This study demonstrated promising therapeutic efficacy with [^161^Tb]Tb-AKIR001 in two preclinical models. Fractionated administration effectively mitigated hematologic toxicity, highlighting it as a potential strategy to increase the therapeutic window. While further evaluation in models expressing normal CD44v6 is highly warranted, our findings support the continued development of terbium-161-based radiopharmaceuticals as a compelling therapeutic approach.

## Supplementary Material

Supplemental material accompanying this article includes a detailed list of used material and reagents, comparison of tumor volumes between groups at treatment start, a biokinetic comparison between [^161^Tb]Tb-AKIR001 and [^177^Lu]Lu-AKIR001, and numeric data from the biodistributions of [^161^Tb]Tb-AKIR001. Histopathological data is available both in the supplemental PDF and as a separate Excel file.

Supplemental histopathological Excel file.

## Figures and Tables

**Table 1:** ** Decay characteristics.** Decay properties of lutetium-177 and terbium-161, adapted from Alcocer-Ávila et al. 8.

	Terbium-161	Lutetium-177
**Half-life**	6.91 d	6.65 d
**Decay mode**	β^-^ (^161^Dy, 100%)	β^-^ (^177^Hf, 100%)
**E_β-,av_ [keV]**	154.3	133.3
**CE (keV per decay)**	39.28	13.52
**AE (keV per decay)**	8.94	1.13
**Total electron energy per decay (keV)**	202.5	147.9
**γ for imaging: energy in keV (% abundance)**	75 (10.2%)	208 (11%); 113 (6.4%)
**Photons X and γ (total energy per decay in keV)**	36.35	35.1

**Table 2:** ** Radiopharmaceutical stability evaluated by ITLC**. [^161^Tb]Tb-AKIR001 stability was evaluated in PBS, mouse serum, or PBS with a 500-fold molar excess of EDTA. RCP measured by ITLC at 1 h, 24 h, and 48 h were compared to the initial yield at 0 h. Data presented as mean ± s.d. from three independent experiments.

Time (h)	50 mg/mL ascorbate			0 mg/mL ascorbate		
	PBS	Serum	EDTA	PBS	Serum	EDTA
**1**	98.7 ± 1.7	98.5 ± 1.4	99.8 ± 0.2	100 ± 3.2	99.0 ± 1.6	98.4 ± 2.0
**24**	98.9 ± 1.9	95.7 ± 3.4	96.0 ± 4.4	99.1 ± 1.4	96.4 ± 3.0	96.3 ± 3.4
**48**	97.3 ± 3.3	91.3 ± 6.2	89.7 ± 8.1	96.2 ± 3.5	82.6 ± 6.2	87.8 ± 2.7

**Table 3:** ** Radiopharmaceutical stability evaluated by HPLC.** RCP of [^161^Tb]Tb-AKIR001 in PBS (N = 1) with or without 50 mg/mL ascorbate measured by HPLC at 4 h, 24 h, and 48 h post-labeling.

Time (h)	50 mg/mL ascorbate (area%)	0 mg/mL ascorbate (area%)
**4**	99.6	99.5
**24**	99.4	92.5
**48**	97.4	90.0

**Table 4:** ** Kinetic data.** Association rate constant (k_a_), dissociation rate constant (k_d_), and equilibrium dissociation constant (K_D_) estimates for [^161^Tb]Tb-AKIR001 binding to A431 and BxPC3 cells. BxPC3 data presented as mean of three independent experiments ± s.d.

Parameter	A431	BxPC3
k_a_ (M^-1^ × s^-1^)	2.4 × 10^5^	2.44 × 10^5^ (± 1.26 × 10^4^)
k_d_ (s^-1^)	3.4 × 10^-6^	1.05 × 10^-6^ (± 1.04 × 10^-6^)
K_D_ (pM)	14.2	4.19 (± 4.12)

**Table 5:** ** Estimated absorbed organ doses derived from *ex vivo* data.** [^161^Tb]Tb-AKIR001 and [^177^Lu]Lu-AKIR001 organ dosimetry estimations with absorbed energy per decay derived from the MOBY platform or Monte Carlo simulations. Based on data from mice bearing BxPC3 xenografts, with lutetium-177-data derived from a previous study 12. Data presented as mean absorbed dose (Gy/MBq) ± s.d. and tumor-to-tissue ratio.

	Terbium-161 (^161^Tb)		Lutetium-177 (^177^Lu)		
Tissue	Absorbed dose (Gy/MBq, mean ± s.d.)	Tumor-to-tissue ratio	Absorbed dose (Gy/MBq, mean ± s.d.)	Tumor-to-tissue ratio	^161^Tb/^177^Lu dose ratio
Bone marrow	0.63 ± 0.08	27	0.44 ± 0.06	26	1.42
Liver	1.18 ± 0.14	14	0.83 ± 0.01	14	1.42
Kidney	0.94 ± 0.15	18	0.66 ± 0.01	17	1.43
Spleen	0.66 ± 0.15	25	0.46 ± 0.01	25	1.44
Lungs	0.62 ± 0.13	27	0.43 ± 0.09	27	1.45
Intestines	0.17 ± 0.03	98	0.12 ± 0.02	95	1.43
Skin	0.60 ± 0.08	28	0.42 ± 0.06	27	1.41
Muscle	0.14 ± 0.02	119	0.10 ± 0.02	113	1.39
Bone	0.47 ± 0.04	36	0.32 ± 0.02	36	1.47
Carcass	0.38 ± 0.05	44	0.27 ± 0.03	42	1.4
BxPC3 tumor	16.7 ± 2.4	1	11.4 ± 1.6	1	1.46

**Table 6:** ** Parameters characterizing treatment efficacy.** Median survival for all treatment groups, including percentage of complete remission and time for euthanasia of the first and last mouse.

	Treatment	First mouse euthanized (day)	Last mouse euthanized (day)	Median survival (day)	Complete responders (%)
**A431**	PBS	4	6	5	0
	5 MBq [^161^Tb]Tb-AKIR001	4	43	20	0
	7.5 MBq [^161^Tb]Tb-AKIR001	22	73	22	0
	10 MBq [^161^Tb]Tb-AKIR001	65	90 ^1)^	Undefined	60
**BxPC3**	PBS	19	40	25	0
	4 MBq [^161^Tb]Tb-AKIR001	56	100 ^1)^	90	12.5
	8 MBq [^161^Tb]Tb-AKIR001	62	100 ^1)^	Undefined	50
	2 x 4 MBq [^161^Tb]Tb-AKIR001	62	100 ^1)^	Undefined	50

1) All remaining mice were euthanized the last day of the study (day 90 for the A431-study or day 100 for the BxPC3-study).

## Data Availability

Data are available from the corresponding author upon request.

## Abbreviations

%IA/g: percent injected activity per gram of tissue
AE: Auger electrons
CE: conversion electrons
CT: computed tomography
DMEM: Dulbecco’s Modified Eagle Medium
EDTA: ethylenediaminetetraacetic acid
HGB: hemoglobin
HPLC: high-performance liquid chromatography
i.p.: intraperitoneal
ITLC: instant thin-layer chromatography
i.v.: intravenous
MIP: maximum intensity projection
PBS: phosphate-buffered saline
PDAC: pancreatic ductal adenocarcinoma
p.i.: post-injection
PLT: platelets
RCP: radiochemical purity
RBC: red blood cells
RPMI-1640: Roswell Park Memorial Institute 1640 medium
SCC: squamous cell carcinoma
s.d.: standard deviation
s.c.: subcutaneous
SPECT/CT: single-photon emission computed tomography/computed tomography
TIAC: time-integrated activity concentration
TRT: targeted radionuclide therapy
WBC: white blood cells
