# Supplementary material for: Preclinical efficacy and dosimetry of CD44v6-directed terbium-161 radionuclide therapy
Source: Theranostics. 2026 Aug 24;16(15):8880–91. doi: 10.7150/thno.135243 (PMC13549377; doi:10.7150/thno.135243)
Supplement: Supplementary file 1 [file thnov16p8880s1.pdf]

Supplemental figures

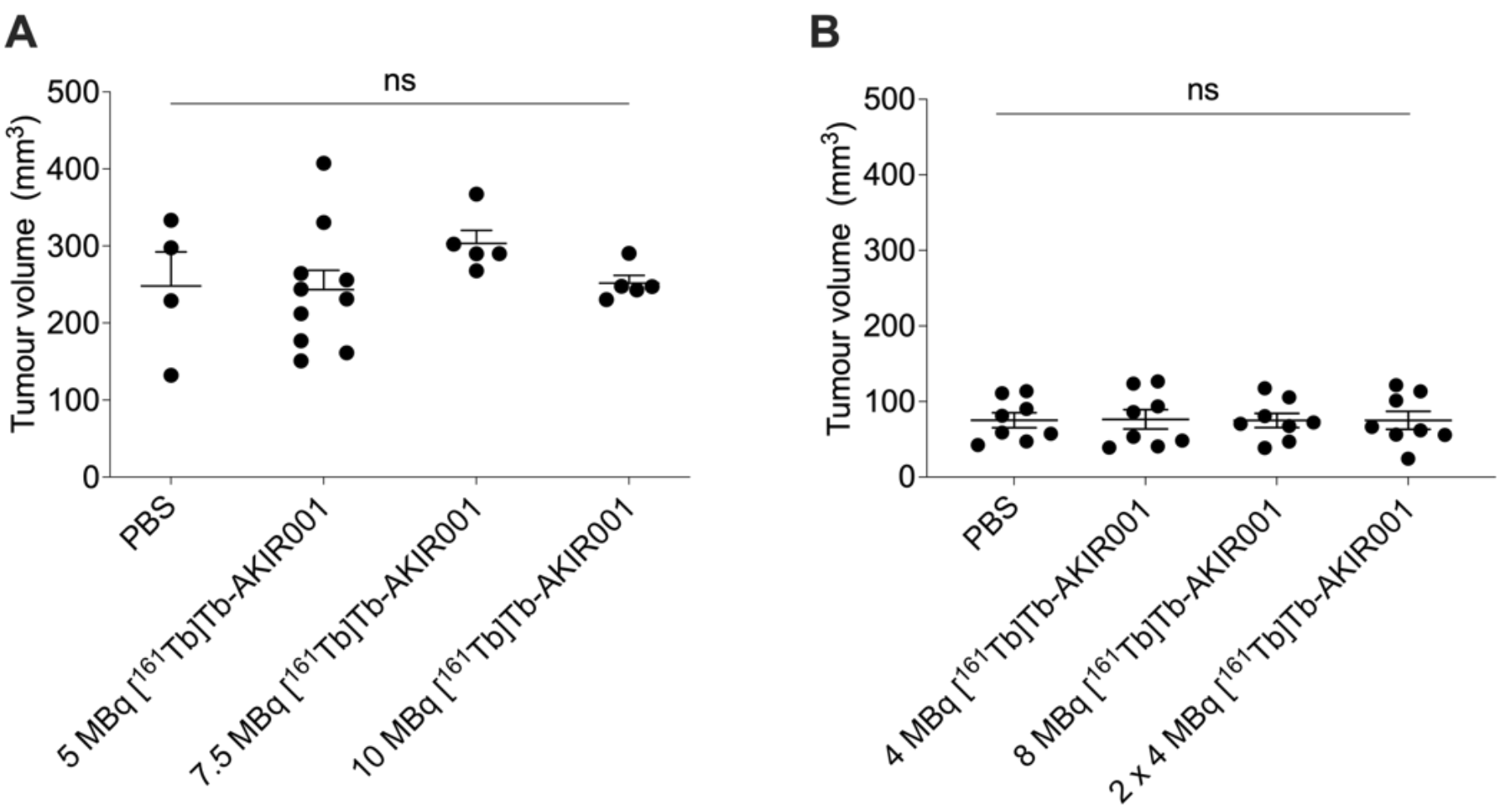

Figure S1. Tumor volumes at treatment start. At day 0 when treatment was initiated, tumor volumes were comparable between groups. (A) A431 and (B) BxPC3. Error bars represent s.e.m.

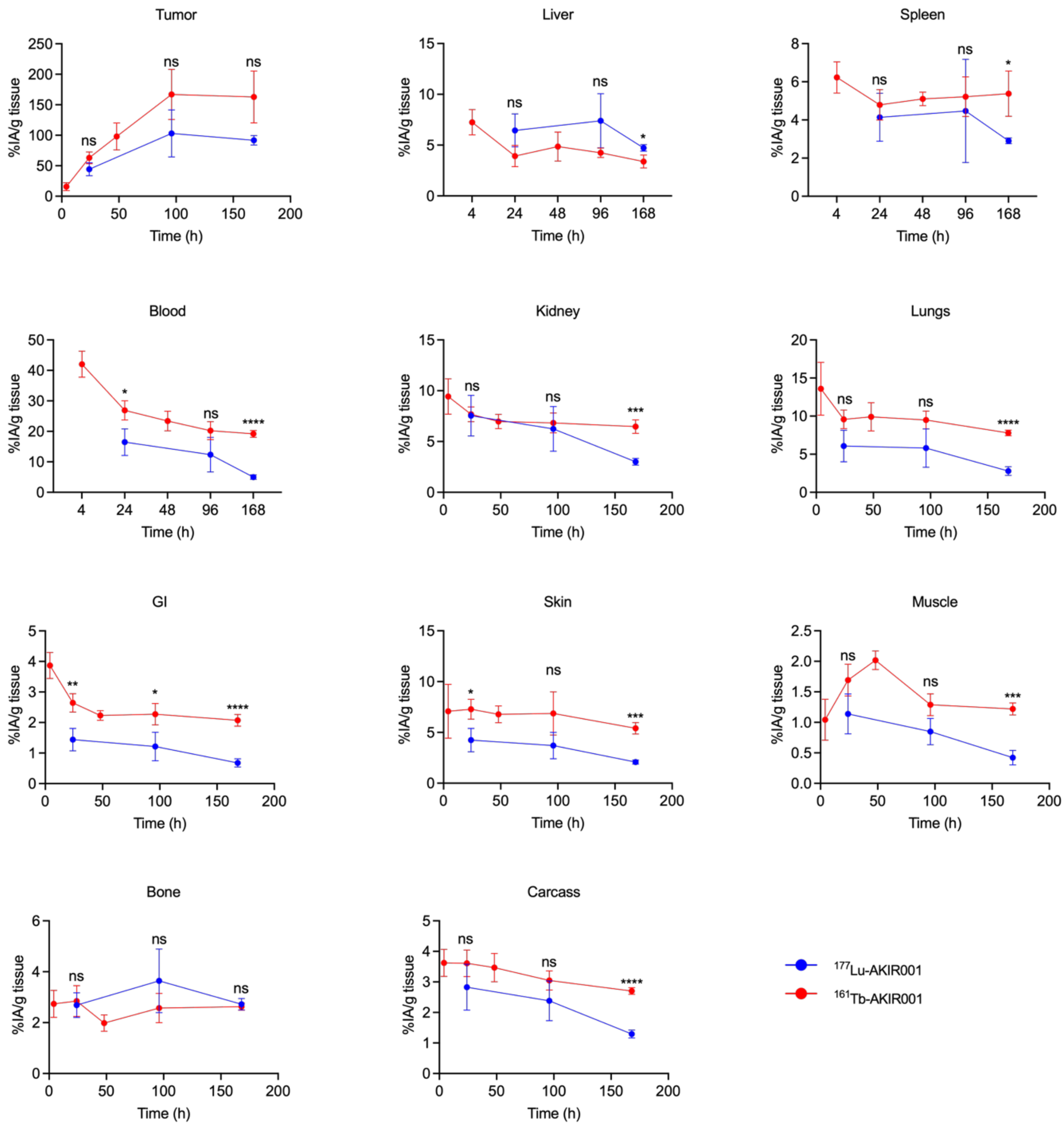

Figure S2. Comparison of biokinetics. Comparison of organ-wise biokinetic data for [^{161}Tb]Tb-AKIR001 and [^{177}Lu]Lu-AKIR001 at 24, 96, and 168 h post-injection. Number of * indicates level of significance. Error bars represent s.d

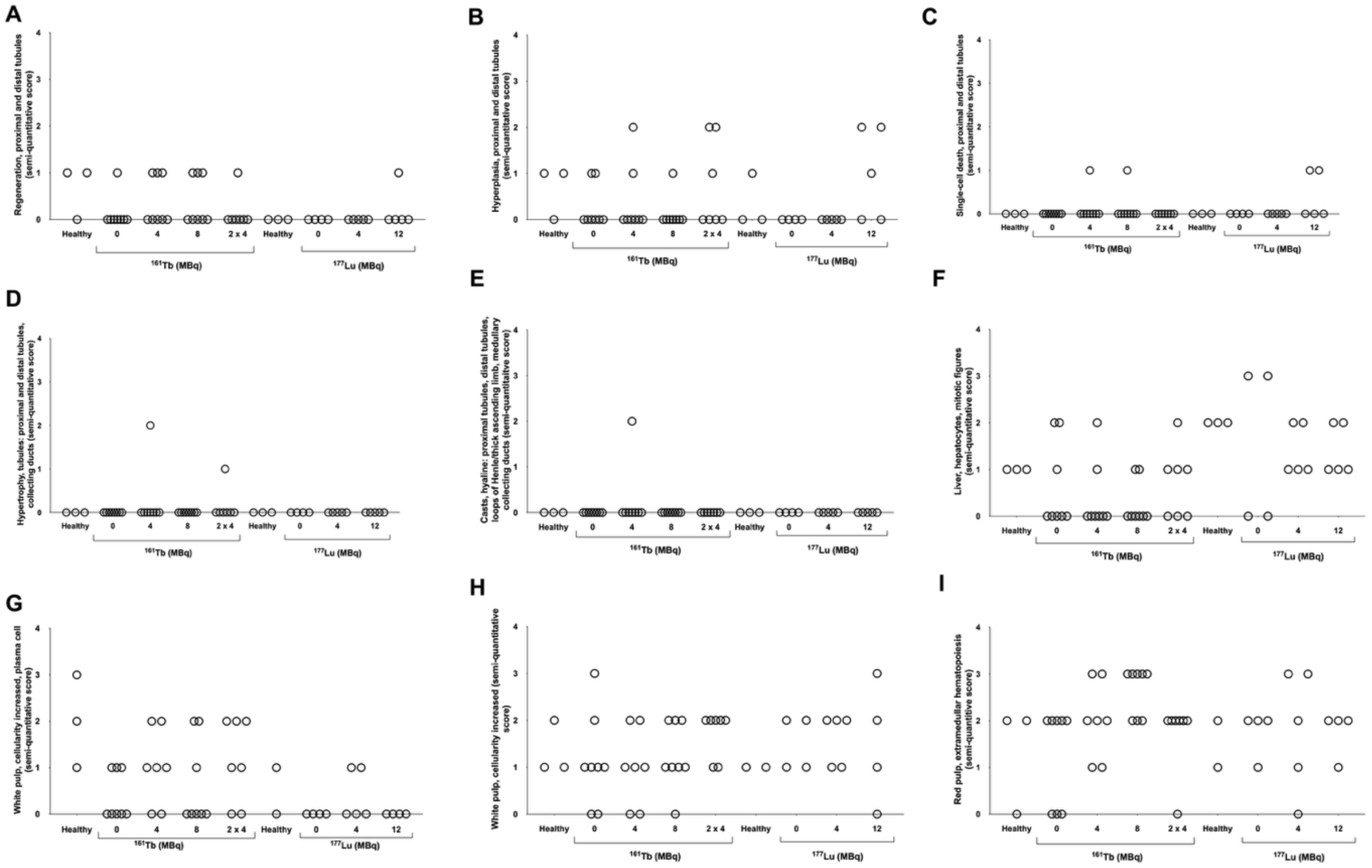

Figure S3. No toxic effects found in kidney, liver or spleen of TRT-treated mice. Histopathological evaluation, semiquantitative scoring of kidney, liver, and spleen. (A-E) kidney, (F) liver, (G-I) spleen. ANOVA analysis according to Kruskal-Wallis resulting in (A) p = 0.2846, (B) p = 0.2287, (C) p = 0.2660, (D) p = 0.7670, (E) p = 0.7168, (F) p = 0.0359 i.e. significant difference within the group, but a post-hoc Dunn's test resulted in no significant differences between groups, (G) p = 0.6488, (H) p = 0.1299, and (I) p = 0.0196 i.e. significant differences within the group, but a post-hoc Dunn's test resulted in no significant differences between groups.

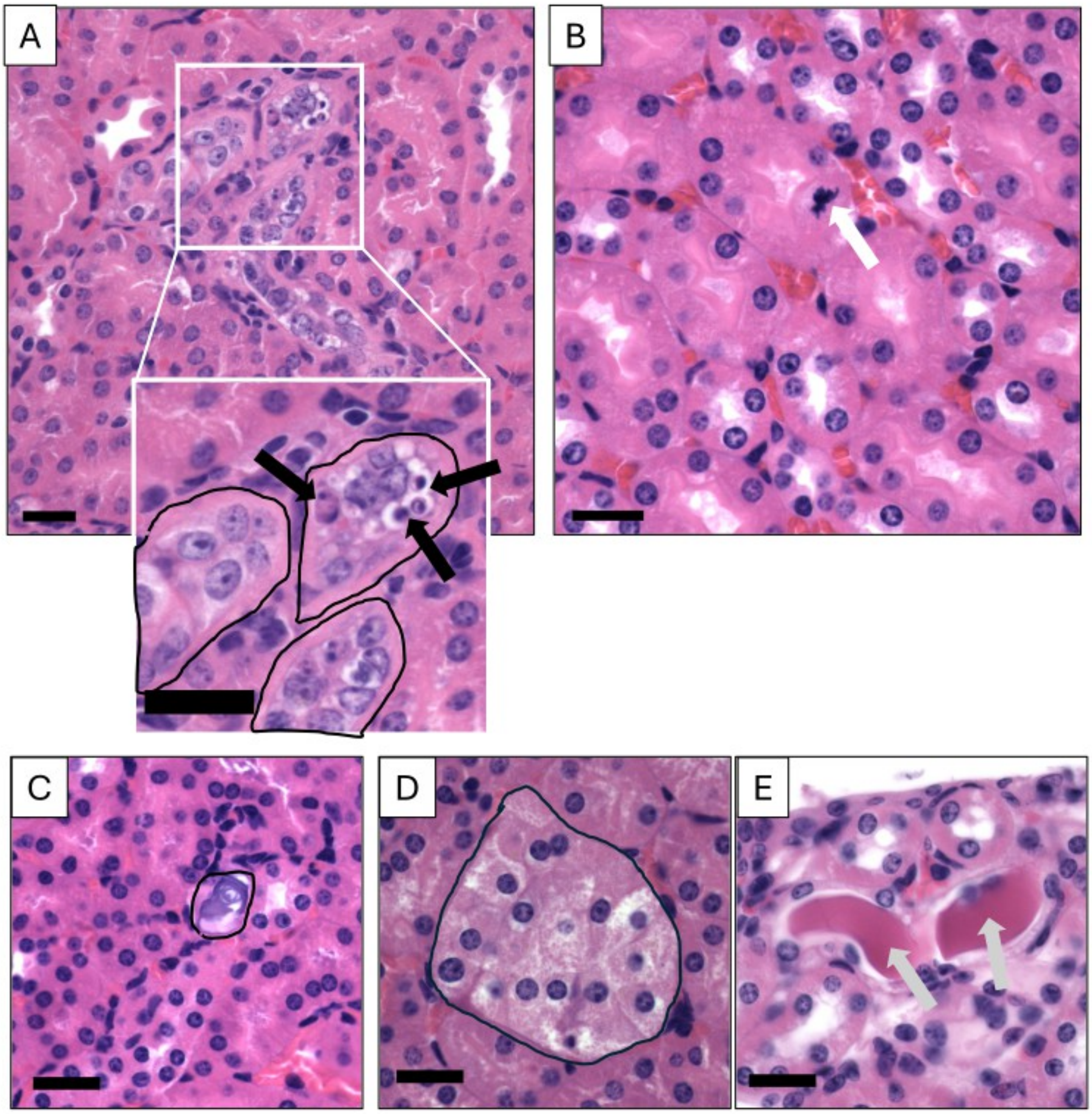

Figure S4. Examples of the six lesions found in the kidneys. Mayer's hematoxylin eosin stain, bar = 20 µm. (A) Mouse 14, treated with ^{177}Lu, 12 MBq: regeneration, tubule (black outline in inset image) and single-cell death of tubular epithelial cells (black arrows). (B) Mouse 13, treated with ^{161}Tb 4MBq: mitotic figure, tubular epithelial cell (white arrow). (C) Mouse 9, PBS-control. Tubular mineralisation (black outline). (D) Mouse 12, treated with ^{161}Tb 4MBq: hypertrophy, tubular epithelial cells (black outline). (E) Mouse 15, treated with ^{161}Tb 4MBq: eosinophilic casts, collecting duct (gray arrow).

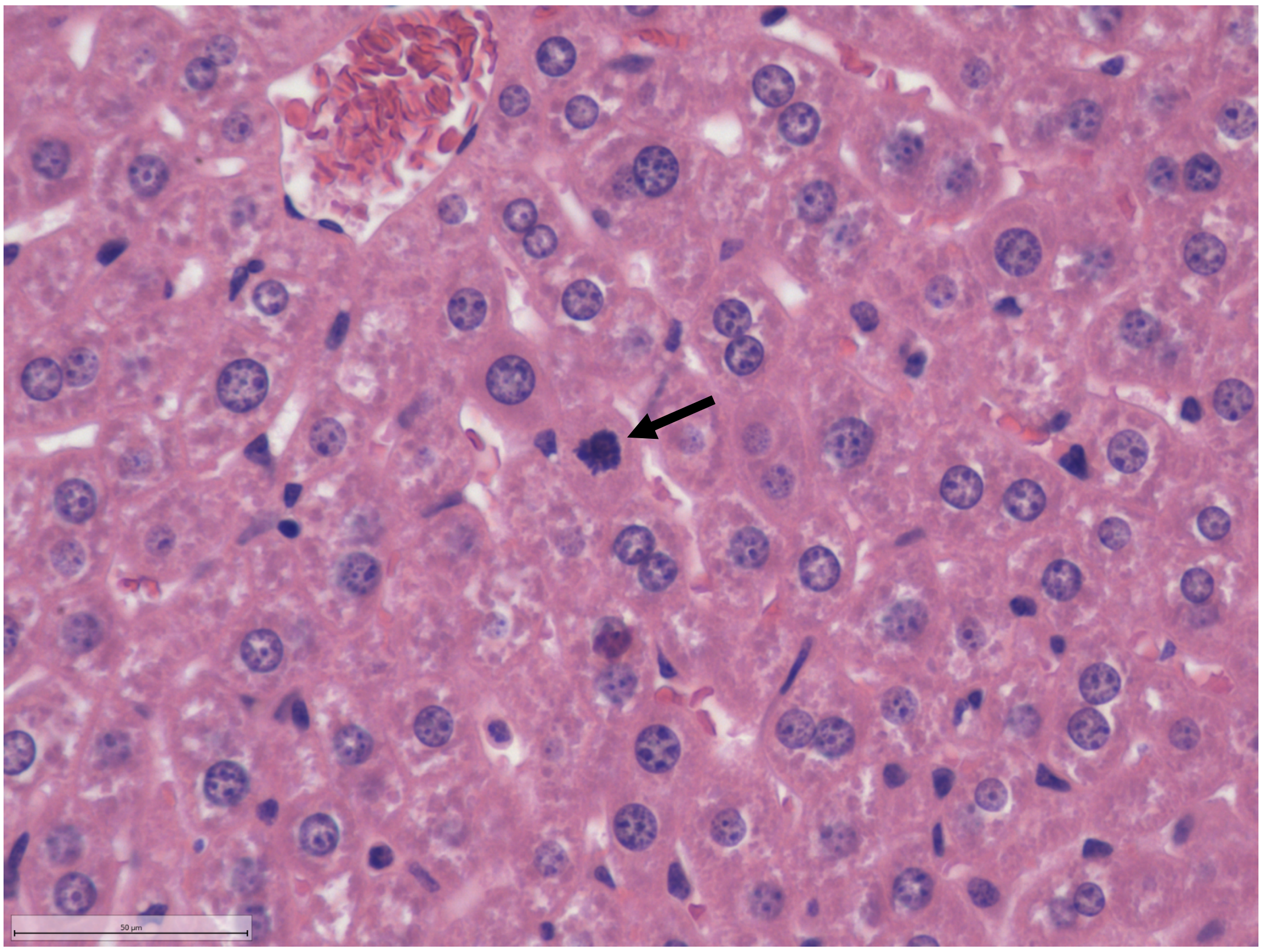

Figure S5. Examples of the single lesion found in the liver. Mayer's hematoxylin eosin stain. Mouse 26, treated with ^{177}Lu, 4 MBq: Mitotic figure, hepatocyte (black arrow). Bar = 50 µm.

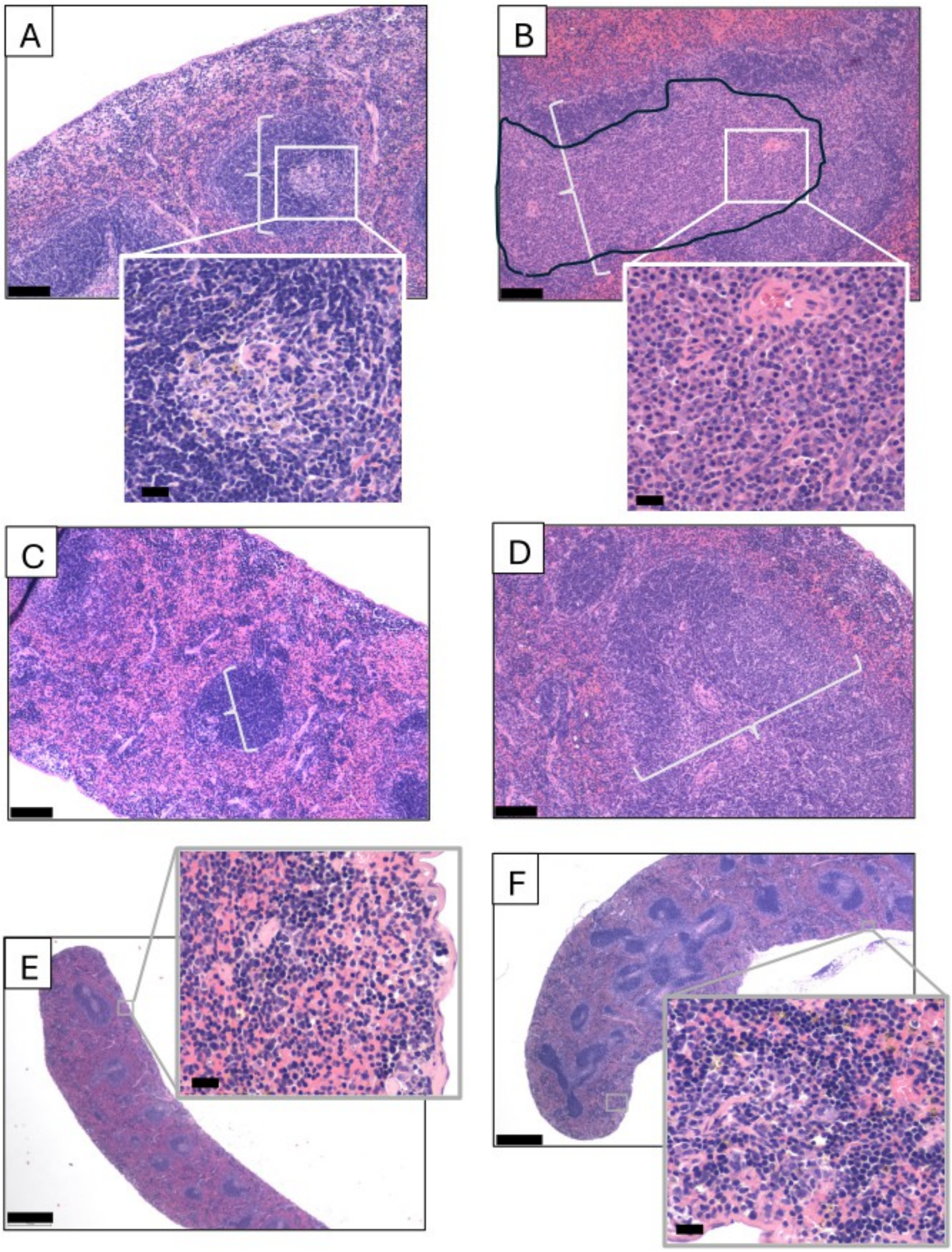

Figure S6. Examples of the three lesions found in the spleen. Mayer's hematoxylin eosin stain. Bar = 100 µm (A-D); bar = 20 µm (insets to A, B, E, F); bar = 500 µm (E, F). Bracket shows white pulp diameter. (A- B) White pulp, increased cellularity, plasma cells, insets show perivascular sheath of white pulp: (A) Mouse 28 of the ^{161}Tb-group, treated with 8 MBq ^{161}Tb, score 0. (B) Mouse 2, healthy control in the ^{161}Tb-group, score 3. Plasma cell infiltrate outlined in black. (C-D) White pulp, increased cellularity. (C) Mouse 10, PBS-treated control in the ^{161}Tb group, score 0, (D) Mouse 4, PBS-treated control in the ^{161}Tb group, score 3. (E-F) Red pulp, extramedullary hematopoiesis, increased, insets show red pulp with hematopoiteic cells. (E) Mouse 8, PBS-treated control in the ^{161}Tb group, score 0, (F) Mouse 30, treated with 8 MBq ^{161}Tb, score 3.

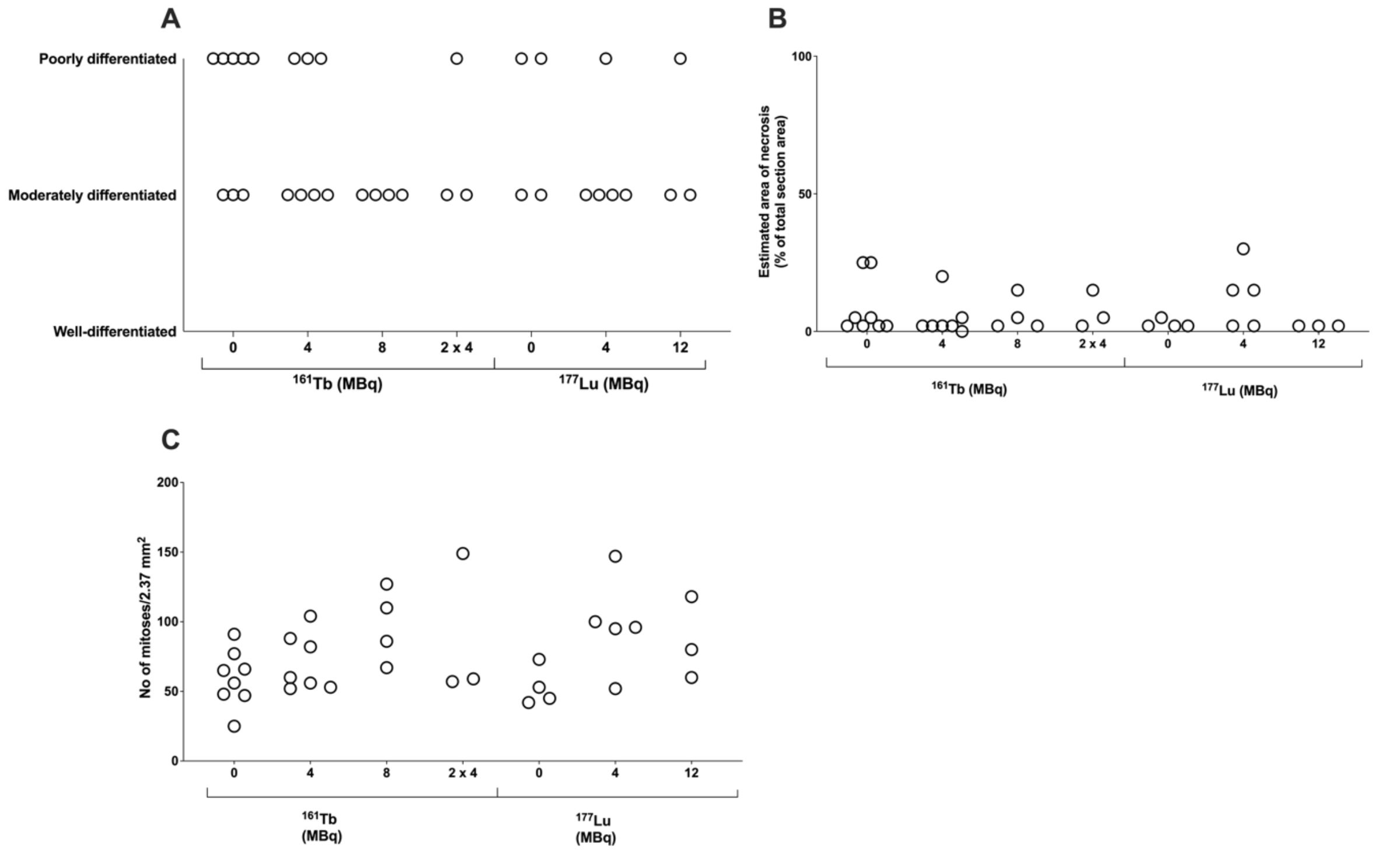

Figure S7. Histopathological evaluation of BxPC3 tumors in BALB/c nu/nu mice treated with TRT. Groups were compared using the Kruskal-Wallis method. (A) No difference in semi-quantitative evaluation of neoplastic cell differentiation, p = 0.4972. (B) No difference in estimated area of necrosis within the examined tumor section, p = 0.4754. (C) No difference in number of mitoses per 2.37 mm^2, p = 0.0729.

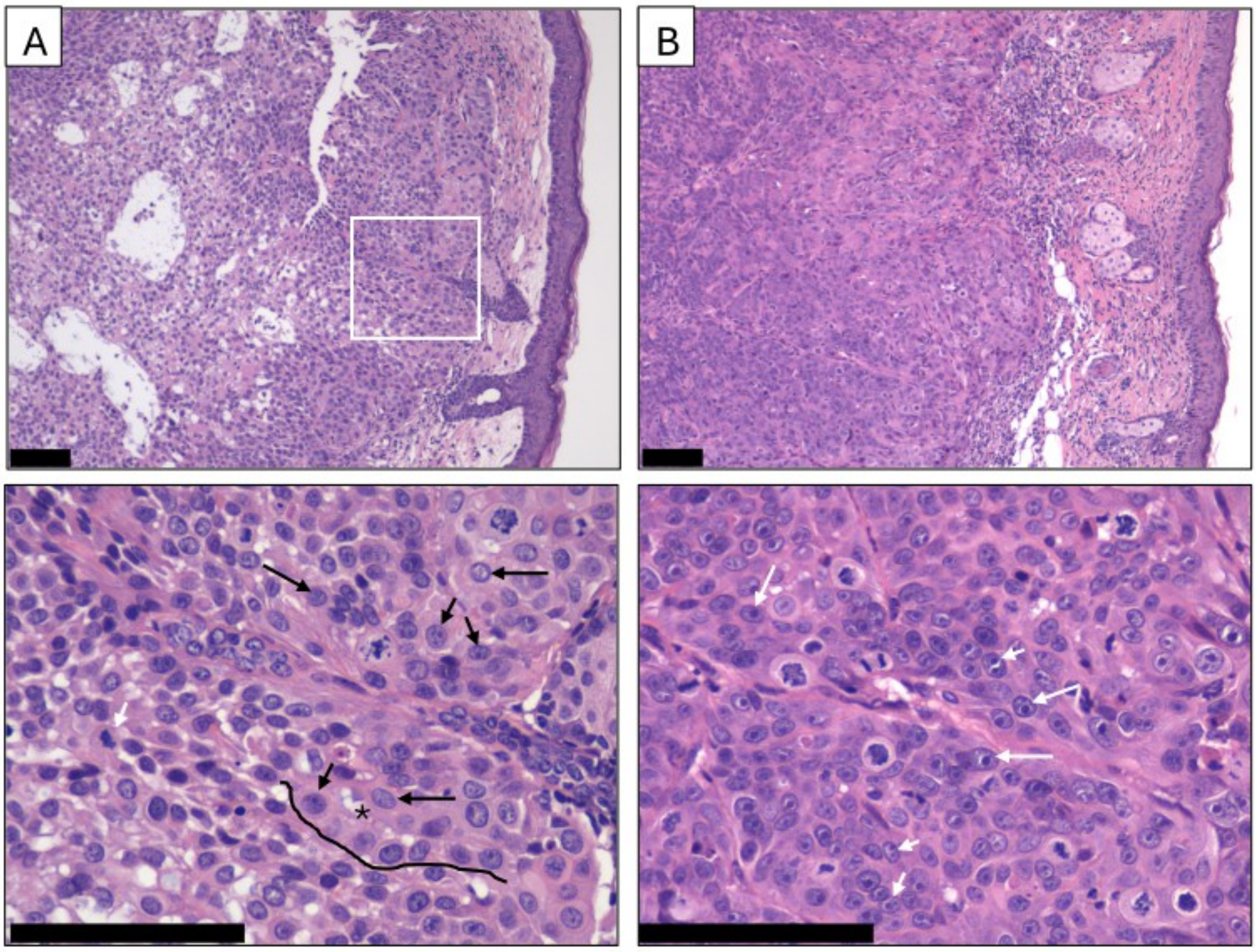

Figure S8. Examples of the histopathological appearance of BxPC3 tumors in BALB/c nu/nu mice treated with TRT Mayer's hematoxylin eosin stain. Bar = 100 µm. (A) Mouse 17, PBS-treated control in the ^{161}Tb-group. Moderately differentiated: formation of acinar structures (* = lumen) and rows (oriented along the black line) can be found. Neoplastic cells have very small, indistinct (long, black arrows) or one or more small (short black arrows) nucleolus. (B) Mouse 12 in the ^{161}Tb-group, treated with 4 MBq ^{161}Tb. Poorly differentiated: no or minimal formation of acinar structures and rows. Neoplastic cells have one (long white arrows) or more (short white arrows) prominent nucleoli.

Supplemental tables

Table S1. Reagents and resources. List of reagents and material, their application and corresponding product information.

Name	Application	Product information
BxPC3	Cell culture	CLS, 305031-061123
A431	Cell culture	ATCC, CRL-1555
RPMI-1640	Cell culture	BioWest, L0500-500
DMEM	Cell culture	Gibco, 31966-021
FBS	Cell culture	Sigma-Aldrich, F7534
Penicillin:Streptomycin	Cell culture	BioWest, L0022-100
Trypsin-EDTA	Cell detachment	Gibco, 25200056
AKIR001	Radiolabelling, treatment	Akiram Therapeutics AB
p-SCN-Bn-DOTA	Radiolabelling	Macrocyclics, B-205
$[^{161}Tb]TbCl_3$	Radiolabelling	POLATOM, RTb-1
Sodium ascorbate	Radiolabelling	Sigma-Aldrich, A7631
Ammonium acetate	Radiolabelling	KEBO Lab AB, 1.1116
Hydrochloric acid	Radiolabelling	Merck KgaA, 1.00314
Metal-free water	Radiolabelling	Honeywell, 95305
Chelex100	Radiolabelling	Sigma-Aldrich, C7901
EDTA	Stability assay	Sigma-Aldrich, E5391
TSKgel G3000SWXL size exclusion column	Stability assay	Tosoh Bioscience, 0008541
TSKgel SWXL guard column	Stability assay	Tosoh Bioscience, 0008543
Acetonitrile	Stability assay	Merck KgaA, 1.13358
Glass microfiber chromatography paper	ITLC	Agilent Technologies, SGI0001
Nunc™ Cell Culture Dishes	LigandTracer	Thermo Scientific, 150350

Table S2. ***Ex vivo*** **biodistribution of [^{161}Tb]Tb-AKIR001 in A431 xenografts.** Mice bearing A431 tumors were injected with 195 kBq/15 µg [^{161}Tb]Tb-AKIR001 and dissected at 4 h (N = 4), 24 h (N = 4) h, 48 h (N = 4), 96 h (N = 7), 168 h (N = 3), and 240 h (N = 3) p.i. Data presented as group means of %IA/g tissue and tumor-to-tissue (T/T) ratio.

Tissue	4 h		24 h		48 h		96 h		96 h (block)		168 h		240 h	
	%IA/g tissue	T/T ratio	%IA/g tissue	T/T ratio	%IA/g tissue	T/T ratio	%IA/g tissue	T/T ratio	%IA/g tissue	T/T ratio	%IA/g tissue	T/T ratio	%IA/g tissue	T/T ratio
Blood	29.6	0.4	12.7	2.2	9.4	3.3	4.9	6.7	11.7	1.3	3.1	9.8	2.7	10.1
Tumor	12.4	1.0	27.7	1.0	31.3	1.0	32.9	1.0	15.2	1.0	30.0	1.0	27.3	1.0
Liver	20.2	0.6	19.3	1.4	10.8	2.9	18.3	1.8	19.6	0.8	13.8	2.2	11.8	2.3
Kidney	8.6	1.4	4.1	6.8	3.7	8.5	2.3	14.4	4.5	3.3	1.7	18.0	1.5	18.3
Spleen	12.6	1.0	16.3	1.7	5.7	5.5	8.8	3.7	11.2	1.4	8.4	3.6	8.1	3.4
Lungs	9.2	1.3	5.5	5.1	4.3	7.3	2.6	12.6	5.2	2.9	1.8	17.1	1.6	16.8
GI	2.1	5.9	1.3	21.6	1.1	28.4	0.5	63.2	1.0	15.4	0.3	98.7	0.4	77.9
Skin	3.6	3.5	3.5	8.0	2.9	10.7	1.4	24.4	2.4	6.2	1.0	28.8	0.7	36.9
Muscle	0.9	14.3	1.0	27.6	0.7	42.0	0.5	61.0	0.8	18.6	0.2	145.9	0.2	119.2
Bone	2.2	5.6	3.0	9.3	1.5	20.3	1.3	26.2	1.9	7.9	1.4	21.2	1.2	23.5
Carcass	2.7	4.6	2.2	12.7	1.9	16.6	1.1	29.7	2.1	7.2	1.0	30.8	1.1	25.5

Table S3. ***Ex vivo*** **biodistribution of [^{161}Tb]Tb-AKIR001 in BxPC3 xenografts.** Mice bearing BxPC3 tumors were injected with 300 kBq/15 µg [^{161}Tb]Tb-AKIR001 and dissected at 4 h (N = 5), 24 h (N = 5) h, 48 h (N = 5), 96 h (N = 4), and 168 h (N = 5) p.i. Data presented as group means of %IA/g tissue and tumor-to-tissue (T/T) ratio.

**Tissue**	**4 h**		**24 h**		**48 h**		**96 h**		**168 h**	
	**%IA/g tissue**	**T/T ratio**	**%IA/g tissue**	**T/T ratio**	**%IA/g tissue**	**T/T ratio**	**%IA/g tissue**	**T/T ratio**	**%IA/g tissue**	**T/T ratio**
Blood	42.0	0.4	26.9	2.4	23.4	4.3	18.7	7.6	19.2	8.4
Tumor	15.8	1.0	63.1	1.0	98.0	1.0	146.9	1.0	162.9	1.0
Liver	7.2	2.2	3.9	17.1	4.9	21.1	4.5	34.4	3.4	48.5
Kidney	9.4	1.7	7.7	8.2	7.0	14.1	6.9	21.4	6.5	25.1
Spleen	6.2	2.5	4.8	13.4	5.1	19.3	5.3	27.9	5.4	30.5
Lungs	13.6	1.2	9.6	6.6	10.0	10.4	9.4	15.5	7.8	20.9
GI	3.9	4.2	2.6	24.0	2.2	43.9	2.4	63.5	2.1	78.9
Skin	7.1	2.4	7.3	8.8	6.8	14.5	7.7	21.2	5.4	30.0
Muscle	1.0	15.9	1.7	37.5	2.0	49.1	1.3	111.3	1.2	132.6
Bone	2.7	5.6	2.8	22.7	2.0	49.5	2.7	57.1	2.6	61.7
Carcass	3.6	4.3	3.6	17.6	3.5	28.6	3.1	47.7	2.7	59.9
